# Health workers' views on quality of prevention of mother-to-child transmission and postnatal care for HIV-infected women and their children

**DOI:** 10.1186/1478-4491-7-39

**Published:** 2009-05-13

**Authors:** Thu Anh Nguyen, Pauline Oosterhoff, Yen Ngoc Pham, Anita Hardon, Pamela Wright

**Affiliations:** 1 Faculty of Public Health, Hanoi Medical University, Hanoi, Viet Nam; 2 Medical Committee Netherlands Vietnam, Hanoi, Viet Nam; 3 Amsterdam School of Social Science Research, University of Amsterdam, Amsterdam, the Netherlands

## Abstract

**Background:**

Prevention of mother-to-child transmission has been considered as not a simple intervention but a comprehensive set of interventions requiring capable health workers. Viet Nam's extensive health care system reaches the village level, but still HIV-infected mothers and children have received inadequate health care services for prevention of mother-to-child transmission. We report here the health workers' perceptions on factors that lead to their failure to give good quality prevention of mother-to-child transmission services.

**Methods:**

Semistructured interviews with 53 health workers and unstructured observations in nine health facilities in Hanoi were conducted. Selection of respondents was based on their function, position and experience in the development or implementation of prevention of mother-to-child transmission policies/programmes.

**Results:**

Factors that lead to health workers' failure to give good quality services for prevention of mother-to-child transmission include their own fear of HIV infection; lack of knowledge on HIV and counselling skills; or high workloads and lack of staff; unavailability of HIV testing at commune level; shortage of antiretroviral drugs; and lack of operational guidelines. A negative attitude during counselling and provision of care, treating in a separate area and avoidance of providing service at all were seen by health workers as the result of fear of being infected, as well as distrust towards almost all HIV-infected patients because of the prevailing association with antisocial behaviours. Additionally, the fragmentation of the health care system into specialized vertical pillars, including a vertical programme for HIV/AIDS, is a major obstacle to providing a continuum of care.

**Conclusion:**

Many hospital staff were not being able to provide good care or were even unwilling to provide appropriate care for HIV-positive pregnant women The study suggests that the quality of prevention of mother-to-child transmission service could be enhanced by improving communication and other skills of health workers, providing them with greater support and enhancing their motivation. Reduction of workload would also be important. Development of a practical strategy is needed to strengthen and adapt the referral system to meet the needs of patients.

## Background

Prevention of mother-to-child transmission (PMTCT) has been considered a simple intervention: just giving medication to prevent viral transmission from mother to child. Now, though, PMTCT is recognized as a comprehensive set of interventions requiring capable health workers. It starts with testing pregnant women for HIV, preferably during their first antenatal visit. When giving the test result, health care workers should provide good counselling, including information about PMTCT options.

The health system should ensure that HIV-positive women receive the PMTCT services that they choose and should provide postnatal care. All along the timeline from finding out their serostatus to getting treatment for HIV-related problems, women and their children should be followed closely. The need for comprehensive and long-term care for HIV-infected women has become a challenge for health systems, particularly where lack of coordination among different facilities is common [1,2].

Viet Nam's HIV epidemic is still in a concentrated phase, with the highest seroprevalence among high-risk key populations, including injecting drug users (IDU), female sex workers (FSW) and men who have sex with men (MSM). Hanoi is one of 10 provinces/cities reported to have the highest number of HIV infections per 100 000 inhabitants. After the first case of AIDS was identified, in 1992, the HIV epidemic in Hanoi increased sharply by 1994. The capital has 12 628 persons living with HIV/AIDS (PLHIV), mostly IDU from poor families. Currently, there are 3623 AIDS patients and 2081 who had died of AIDS in the city. Although HIV is predominantly concentrated among IDU and FSW, it is gradually spreading among the general population. In 2007, HIV prevalence among pregnant women attending antenatal care (ANC) clinics in the Hanoi was 0.34% [3].

Hanoi was selected as the study site because comprehensive PMTCT care is theoretically available there. Hanoi had 45 hospitals, 290 surgeries and five international hospitals. The national hospitals in Hanoi serve as referral centres for the northern half of Viet Nam. HIV testing and counselling for pregnant women are offered at health facilities at district or higher level, but often only after the 28^th ^week of gestation. HIV-positive women are referred to provincial or national hospitals for ARV prophylaxis, delivery and postnatal care. Hanoi health services have sufficient supplies of prophylactic ARV to meet the demand. Antiretroviral therapy for adults is available at district level or higher. HIV-exposed infants are offered polymerase chain reaction (PCR) testing and free infant feeding formula for at least six months, while free paediatric ARV is available for children three years of age or older.

The extensive health care system in Hanoi reaches the commune level, but multisectoral and cross-programme collaboration to link the pillars of the World Health Organization's (WHO) comprehensive approach to PMTCT are weak [4]. For example, there is little collaboration between the programmes for HIV/AIDS and family planning. Our previous work suggested that a large number of HIV-infected pregnant women remain undetected by the health system [5]. In addition, a number of barriers result in failure to access PMTCT during pregnancy and delivery [6-11]. Among the weak points identified were that HIV-infected women received inadequate information about postnatal care, but even when they had knowledge, many expressed fear of stigma and discrimination that reduced their access to care; HIV testing is not available via health services at commune level, where many pregnant women go for care and delivery; and women feared lack of confidentiality of HIV test results [4,12].

Our previous studies on the experiences and views of women about the provision of PMTCT in Hanoi included criticisms about the quality of services provided by health workers [4]. Other studies in Asia found that health workers were unwilling to provide appropriate care for HIV-positive pregnant women, often because of their own fear or lack of knowledge, or because of high workloads and lack of staff [13,14]. Inadequate health care delivery may be caused by a variety of factors, but we need to identify the main issues before planning interventions to strengthen it.

We report here the health workers' perceptions on the factors that lead to their failure to give good quality PMTCT. The findings should inform the development of a more effective programme for the fourth prong of the WHO-recommended comprehensive PMTCT programme.

## Methods

Sampling of study sites was based on availability of services in Hanoi and their level and function in the health care system. All hospitals in Hanoi that provide ARV, PMTCT and opportunistic infections (OI) treatment were selected, including all health facilities providing HIV testing. Additionally, all health facilities in one high-prevalence and well-resourced district were selected. In that district, interviews were carried out at all levels of the health system involved in PMTCT: district health centre, district maternity ward, district committee for family planning and mother and child health, and preventive medicine centres, including HIV testing sites. The research team also visited commune health stations in the district. Details are presented in Table 1.

**Table 1:** Health facilities in the study

**Level**	**Service**	**Number**
National	PMTCT	1 National Obstetric Hospital

	HIV paediatric treatment	1 National Pediatric Hospital

	ART for adults	1 National General Hospital

Provincial	PMTCT	1 Hanoi Obstetric Hospital

	Paediatric care	1 Saint Paul Hospital (provincial general hospital)

District	ART for adults	1 Dongda Hospital

	HIV testing and ANC care	1 Dongda Health Centre

	Primary health care, including ANC and immunization	2 Commune Health Stations

**Total**		**9 facilities**

Selection of respondents was based on their function, position and experience in the development or implementation of PMTCT policies/programmes. Interviewees were first screened to check that they had appropriate positions and at least one year of experience with PMTCT, so that they could provide insightful information. They included doctors, nurses, midwives, counsellors, laboratory technicians and programme managers. Detailed information on interviewees is presented in Table 2.

**Table 2:** Number of interviews by type of health staff

**Type of health staff**	**National level**	**Provincial level**	**District level**	**Commune level**	**Total**
Programme manager	4	2	4	0	10

Doctor	5	3	4	4	16

Nurse	2	2	2	2	8

Midwife	0	0	2	4	6

Counsellor	5	4	4	0	13

**Total**	**16**	**11**	**16**	**10**	**53**

We conducted semistructured interviews with these 53 health workers about their experience in implementation of services for PMTCT, their point of view about users of their services and their perception about the challenges they faced in providing good PMTCT services in their health facility.

In addition, unstructured observations were made in nine health facilities, in waiting rooms, counselling rooms, ANC examination wards, delivery wards, postnatal wards, outpatient and inpatient clinics for ARV and OI facilities, and laboratories.

The interviewers were four trained public health and social science researchers. Institutional ethical approval was obtained from the Scientific Committee of Hanoi Medical University and written informed consent was obtained from all interviewees, who were invited to participate voluntarily. The interviews were conducted privately and anonymously. A code book was developed focusing on key findings and terminologies. The transcripts of the semistructured interviews were coded, entered and analysed by means of N-VIVO software.

## Results

### External and internal factors of competence that limit health workers' ability to provide good quality service

Many hospital staff explained their reasons for not being able to provide good care; the most frequently heard was high workload. Observation confirmed that the national and provincial hospitals' ANC caseload was high: the wards were always crowded. One of the main obstetrics hospitals provides ANC to between 200 and 400 pregnant women daily. None of the health facilities offering PMTCT services had recruited additional staff to provide counselling. However, the workload in ANC facilities at district and commune level was not so high, according to the respondents.

"How can I counsel all of the hundreds of women who come every day?" Counsellor, ANC national hospital

"There are many women coming here for ANC and delivery. We do not have enough staff to provide services for them. How can we provide service for HIV-infected women? Even if we want, who will invest for a new infrastructure which has separate area for HIV-infected women?" Manager, ANC provincial hospital

"I have not only this work [providing treatment for HIV-infected patients]. I have to provide treatment for other patients [HIV non-infected patients] and a lot of other work. Very tired." Doctor, ARV district hospital

Another reason given was lack of knowledge regarding PMTCT and lack of skills to provide counselling. Health workers at all levels revealed that their knowledge and skills on counselling are limited.

"There are only very few health staff with only basic information on counselling in this hospital. Poor knowledge and skill is common problem here." Counsellor, ANC provincial hospital

Especially at district or lower level, knowledge is limited regarding ARV prophylaxis and on follow-up care, such as continuing replacement feeding (RF) supplies, infant testing and services for HIV-infected mothers and exposed infants. Consequently, health workers cannot provide adequate counselling on these issues before women are discharged.

"What I can do is to provide information about her HIV test result. I know that there is a medication to prevent transmission of HIV from mother to child, but I don't know exactly. Our common practice is to refer her to provincial hospital." Midwife, ANC commune level

The staff in most health facilities reported having had limited training on PMTCT in general and on counselling in particular; that they apply knowledge and skills gained from observing colleagues conducting counselling sessions; and that refresher courses are rare. The duration of the training varied from two days to two weeks. After the training, counselling is added to their regular ANC or maternity work.

"What we do now is only to inform. [Counsellors] lack knowledge. If they will be trained, who will train them?" Manager, ANC provincial hospital

"We counsel from our experience. To me, our counselling may be not complete. We don't even know what might be our shortcomings". Nurse, ANC commune level

An important point of the comprehensive PMTCT approach is that HIV-infected women should be provided with several different services – such as ARV prophylaxis, formula, counselling and HIV testing for exposed children – provided by different facilities. However, there are no inter- or intra-hospital linkages to make the PMTCT comprehensive. For example, family planning services at a national obstetric hospital are not linked to other departments, including the infectious disease department that provides ANC and delivery services for HIV-infected women. Women are seldom referred to ARV sites for clinical staging or immunological assessment. Referral to postnatal care and social support for both mothers and children is not available at the hospital exit point.

"There is no linkage with obstetric hospitals; they never directly inform us. It is very difficult to know which HIV+ patient or child has been referred to this paediatric hospital for follow-up by what hospital. We don't treat the mothers here. We only provide counselling to them. Support groups? There are some but we don't know where they are." Doctor, paediatric ARV national hospital

Many health workers stated that their task is to provide services available in their own facilities but not services provided by other departments or facilities.

"Well, it is the first time I hear about opportunities for infection prevention for children of women with HIV. That's none of my business." Doctor, ANC district level

"It is not our duty to tell clients about family planning methods. There is a family planning counselling centre over there [points her finger]." Nurse, ANC national hospital

Some respondents complained about the lack of some services. For instance, HIV testing is not available at commune level, so commune health workers cannot provide HIV testing.

Lack of medications was another reason given for not being able to provide services for HIV-infected women. In many health care facilities, the ARV was not consistently available. Often even single-dose Nevirapine (NVP) was lacking, for women who were tested only at the time of labour. Even in the two PMTCT sites in the city, shortage of ARV for adults was observed to happen every few weeks. One problem with the NVP for children was that it is provided as a large bottle (200 ml) of syrup. Once it has been opened, it cannot be kept for long, but very few HIV-exposed children were identified each day. That means that each bottle was not fully used, and that later, drugs were lacking when supplies ran out.

"In some periods, there is a shortage of SD-NVP for PMTCT. We could not do anything in that situation. In practice, the NVP syrup for children is very inconvenient to use. On one day, we have no more than two children to treat; we have to open one bottle for them and the rest of the syrup is unused. The syrup quickly runs out, and then we don't have medication for another child." Manager, ANC provincial hospital

"We also counsel them to use condoms. If someone asks me what they should do to avoid unwanted pregnancy then I tell them. But I do not have condoms to give them for free for family planning." Counsellor, ANC district level

Another issue is that there are no national guidelines on counselling and testing. Observation showed that facilities at provincial and national levels had counselling and PMTCT guidelines and protocols developed by the projects that support those facilities, but most facilities at district or lower level do not have guidelines or even access to them.

Moreover, health workers at all levels often complained about the lack of attention to the needs of health workers when they have to work in a high-risk environment.

"Among 1,000 health workers, how many want to provide care and treatment for HIV-infected patients? There is no good compensation regimen to support staff working with HIV-infected patients. There is no benefit to save the life of patients in the late period, so how could we be enthusiastic?" Doctor, paediatric ARV national hospital

"We receive extra pay for providing treatment for HIV-infected people. But it is just for one health staff while all [12] staff in my department provide service. We have to share among us." Doctor, adult ARV district hospital

### Dual fear among health workers: fear of infection and fear of "problem clients"

#### Fear of infection

Many respondents admitted that they were afraid of HIV transmission from patients, either because they feared being injured by the patients or through an occupational accident, because they lack protective equipment. Observation at adult and pediatric ARV sites supported this finding.

"No one says by words although people may feel fear in their hearts." Doctor, ANC provincial hospital

"That is normal psychology of human beings. Everybody is afraid of AIDS." Nurse, paediatric ARV national hospital

"We cannot be sure [we are not being infected with HIV] when we practise. My husband is a surgeon and has operated on many HIV-infected patients. We go for HIV testing every six months. Not all health workers dare to take [HIV] tests. We try to protect ourselves but there may be accidents that we cannot predict. So we are very frightened." Doctor, ANC district level

"Among staff in this hospital, very few want to take care of HIV+ patients because they fear transmission. If they don't get HIV, they may get tuberculosis. Many of them [health workers] are not yet married." Doctor, adult ARV district level

Besides their worry about the risk of infection for themselves, health workers are also concerned about how their family, their husbands and children may respond when they learn that they have worked with these patients.

"I told my husband that I treated an HIV-infected child with acute diarrhoea and he told me to stay away from him. We worry about scratches in my hand or other small injuries that would allow contact with the virus when we handle bodily fluid such as sputum, stool and blood." Doctor, paediatric provincial hospital

Health workers in departments that do not provide services for HIV-infected patients may be afraid to have close contact with health workers who do provide these services. Frequently, only doctors with no other choice will work in a department of infectious diseases, given all the risks.

"After knowing [we are midwives], other staff run away from us as if we're lepers." Midwife, ANC national hospital

"Not all health workers want to work in this department [infectious diseases]. You can have risk of infection with many diseases. I don't want to work here, but I was assigned by the manager. I have no choice. My family is not happy with my work. In the old days, many female doctors in this department could not get married or got married very late because of their position." Doctor, ANC provincial hospital

Despite the fact that the hepatitis B prevalence in Vietnam is much higher than HIV prevalence, health workers seem to be less afraid of getting infected with hepatitis B. Many of them were vaccinated against hepatitis B and a hepatitis infection does not carry obvious signs or social stigma.

"We were all vaccinated for hepatitis B, so we are not as worried as when we think about HIV." Midwife, ANC commune level

"Hepatitis B, although incurable, has different transmission routes. People who died of hepatitis B are not many or died without knowledge of their infection status. HIV, on the other hand, still frightens people; those who died of HIV have many signs that are obvious." Nurse, ANC provincial hospital

This fear may be partly due to incomplete knowledge and understanding among health workers about HIV, and about the routes and ease of transmission.

"Health workers do not have in-depth understanding about this disease, that's why they are so afraid. I often say to my colleagues that if health workers still fear HIV, when will be able to eradicate the fear in the community?" Doctor, adult ARV site

"Maybe one day, when scientists discover that HIV can be transmitted in another way, we may find out we are already infected." Doctor, PMTCT site

Health workers confront their fear of infectionTo reduce both fear and risk of infection, health workers often find ways to protect themselves, either by trying to identify which patients might be infected with HIV, by using protective equipment, or by avoid exposure to patients as much as possible.

"It is easier for us to prevent transmission if we know who among the patients is infected with HIV." Midwife, ANC provincial hospital

Observation revealed good practice of precaution when health workers assisted deliveries for HIV-positive women, but not always for HIV-negative women. All health workers said they knew how to protect themselves against occupational exposure to HIV and did so very carefully if they knew who was infected with HIV.

"Staff wear protective uniforms, maintain all hygiene practices and disinfection procedure on all equipment used." Nurse, adult ARV district level

However, even if health workers want to protect themselves by using protective equipment, not all health facilities can provide these means for them.

"Health workers do not have enough protective clothes. We have to use cloth coats and short gloves when assisting deliveries for HIV-infected pregnant women. So we often wear a raincoat on top of the cloth coat." Nurse, ANC provincial hospital

Although hospital managers reported that occupational exposure is rare, among the study population we found five health workers who claimed to have had an exposure to blood that they thought might have put them at risk for HIV infection, either because of needle-sticks or blood that went to their eyes. They all informed us that ARV prophylaxis for prevention of occupational exposure is free of charge at an ARV site at district hospital. But only two of them took medication because the others turned out not to need prophylaxis after closer assessment of their exposure.

Prevention of HIV transmission became a good excuse for health workers to avoid taking care of HIV-infected women, or if they had to provide care, to isolate the HIV-infected women for easier control and management.

"We offer counselling, family planning, nutrition, delivery and care after delivery at home, vaccinations for tetanus. We offer this for normal pregnancies including those with hepatitis B. Women who have high risk with HIV are referred. It is not our business." Doctor, ANC site

"Not everyone understands thoroughly about stigma and dread. The more they know, the more they fear and they try to push responsibility to others." Manager, PMTCT site

Many hospital managers emphasized that although the number of HIV-infected women attending their hospitals has been increasing, the number may still account for quite a small number of women in community.

Observation at all the health facilities revealed that HIV-infected women were often placed in a separate room or area. Even at a high-level hospital, where two or three patients often had to share one bed, there was still an empty bed in the room for HIV-infected patients. The manager in an ANC facility explained:

"HIV is an infectious disease, more severe than hepatitis B. Therefore patients should be controlled carefully to avoid transmission to other patients and staff."

#### Fear of "annoying clients"

Not only fear of HIV infection influenced the quality of care provided to HIV-positive women. Some of the weaknesses in providing service were related to the views of health workers regarding HIV-infected people, who are often seen as drug users or sex workers, or as having a "strange appearance". The real or expected behaviour of such people also induces fear and other negative emotional responses in the health workers.

"They often have tattoos and never dress well. There are spots on their arms. Easy to recognize their bluish black lips." Doctor, ANC national hospital

"They inject in our department. How can we have a good attitude toward them?" Nurse, ANC national hospital

Health workers did admit that not all HIV-infected people behaved badly towards them. However, a few bad experiences could give all staff a negative attitude about HIV-infected people in general.

"Almost all HIV-infected patients cannot be trusted. For instance, when they know they have opportunity to have care and support, they often find ways to get as much as possible. Or if they want to leave the hospital, they often lie to the nurse that the doctor already agreed. Or when they have to pay the hospital fee, they often tell the lie that they will pay tomorrow but after that they disappear." Nurse, paediatric ARV national hospital

"When you have to work with them [HIV-infected patients], you will see the difficulties. It's already hard to gain trust from normal patients. Now we have to serve the very scoundrel social class and at the same time, we receive very low salary. We have to provide service because it's our responsibility but we are not happy because they [HIV-infected patients] are drug users, they are very rude. My experience shows that health workers should not be too good to HIV patients." Doctor, ARV district level

Some of these attitudes are based on real experiences, but many are also based on prejudicial expectations, and women wishing to access PMTCT services will be victims of that stigma, too.

#### Perceived role in improving quality of care

Most of the health workers agreed that the quality of care could be improved by several interventions addressing both individual and structural issues.

Reducing workload and providing better compensation for working with risks were mentioned by almost all health workers at provincial and national level as important solutions. In the interviews, hospital managers suggested that it is very difficult to hire new staff because of the limited budget allocated from the government. A better solution would be to rearrange the services in a more logical structure, for example, to replace individual pre-test counselling (which is often not offered anyway) by group counselling, to use peer counsellors to provide counselling and follow-up of care (reducing the burden for health workers and improving the quality of counselling) and to improve the quality of services at a lower level to reduce the burden on the higher levels.

"If we have more equipment, we can deal with our workload with even few staff. For instance, if we are provided a video, leaflets on HIV and PMTCT, and a room with table and chairs, we can do group counselling for pregnant women." Manager, ANC national hospital

"I have seen in a hospital in Thailand that peer counsellors can work in hospital to help health workers doing some administration work, providing counselling, making appointments. We may need to think about how to apply their experience." Manager, ARV district level

However, many doctors and nurses are still unconvinced about the involvement of peer counsellors in the health service, because they feel that peer counsellors do not have enough capacity and the appropriate attitude to do this work, or even bring the potential for crime and corruption into the hospital.

"You can find good peer counsellors in other countries but not in Viet Nam. They have low education. They may become drug dealers or whatever, we don't know." Doctor, paediatric ARV national hospital

Another possible intervention is training and updating information for health workers. Midwives and nurses said they needed to improve their basic knowledge on HIV/AIDS and PMTCT because they received less training than doctors, while doctors preferred to have advanced PMTCT training. All of them asked to be updated routinely on PMTCT.

"Although training has often been provided to doctors but not nurses, in fact training should be conducted more often for both of them." Doctor, paediatric ARV national hospital

Regarding the system, hospital managers admitted that much needs to be done to improve the quality of service – for example, improving ARV procurement, developing detailed PMTCT guidelines taking into consideration the staff's high workload, increasing availability of high technology equipment and providing sufficient protective equipment for health workers.

"Although ARV is sufficient for all HIV-infected women in Hanoi, in the provincial hospital, there are some periods when they lack medicines. The provincial hospital should make a plan on how much medicine they need. The procurement facility should make administrative procedure short and easy for hospitals to get the medicines." Manager, ANC national hospital

"We have implemented PMTCT programme for more than five years without a PMTCT guideline to instruct us. I think a PMTCT guideline should be developed with involvement of all planners and implementers." Manager, ANC provincial hospital

Improvement of the referral system is seen as an important task that needs to be addressed in the near future. Hospital managers propose to have regular meetings of the health network that provides different services for HIV-infected people.

"When there are not a lot of patients, I think it's appropriate to divide tasks among different hospitals. But we also need to link all hospitals. In practice, the linkage is loose: that makes difficulties for patient access to services. It is due to lack of information about services provided by other places, lack of active coordination to link between different services, people are too busy to think about other services besides the treatment that we can provide. Some health workers are not aware of the need to refer patients, or if they do, patients don't want to go and disclose their positive status in other hospitals, or may not go even because of lack of referral forms." Manager, ARV district hospital

Some health workers also proposed that all services should be offered at one point for better coordination.

"Now we have Global Fund project providing ARV at district health centre. Why don't we provide PMTCT and paediatric treatment at the same facility? I have also heard that there are some self-help groups working at district level that can provide further support for HIV-infected people. The Ministry of Health should think about how to make one facility able to provide all services. That could help to avoid loss to follow-up, which is common among HIV-infected people." Manager, ANC national hospital

The health workers did perceive not only problems but also solutions and seemed to have some motivation to solve the problems and to provide better services for HIV-positive women seeking PMTCT services.

## Discussion

Studies in Viet Nam have demonstrated that both HIV-infected and non-infected women had many criticisms of ANC and delivery services, about provision of information about PMTCT and counselling, and about stigma displayed by health care workers [3,15]. At present, the health service has not yet addressed this gap. Access to comprehensive PMTCT is still very poor, even in such a well-resourced setting as Hanoi [16]. Because the health care workers are subjected to many accusations about their performance in this context, this study was undertaken to find out their opinion regarding these gaps and weaknesses.

Health care workers usually want to do a good job and provide good care for patients. However, they are often unable to provide as good care as they would like, particularly in facilities with an overload of clients [17,18]. Results of a survey among women who had been pregnant in Hanoi revealed that they attended and paid for ANC services at higher-level health facilities (provincial and district hospitals) rather than go to commune health stations where ANC services are free of charge [4]. High workloads were observed at provincial hospitals, while district hospitals and commune health stations appeared to have more time and space for pregnant women. Studies of ANC services in Vietnam have identified a number of weaknesses. Staff shortages and staff motivation can significantly affect the quality of service, especially for counselling, which takes a long time to do well [12,19-22].

In the case of HIV and PMTCT, additional reasons for the unsatisfying performance included inadequate knowledge and skills due to lack of training, medicines, protective equipment and practice guidelines. Health care workers had poor knowledge about HIV and about prevention of occupational exposure to HIV, especially at district or lower levels. Even in the expected sources of expertise, the medical schools around Vietnam, 70% of medical students and 48% of lecturers recapped used needles by hand, while two thirds always cleaned their hands with antiseptic after contact with blood. Sixty percent of medical students and 37% of lecturers had been exposed directly to blood or body fluids and were worried about HIV transmission. However, 15% of the respondents recommended antibiotics for post-exposure prophylaxis, while one third proposed ARV prophylaxis [23]. These results reveal a disturbing lack of knowledge and awareness about HIV, even among the medical profession. Lack of practical needs can become an excuse for health care workers to justify their fear of HIV infection and their reluctance to provide good services for HIV-infected people [7,24].

As the HIV epidemic has evolved in Viet Nam, both governments and international donors have given priority to prevention and surveillance activities. The main reason is that Viet Nam has had success up to now using surveillance and containment to control infectious diseases such as polio, SARS and, more recently, avian flu. HIV/AIDS policy and practice also aims foremost at controlling the spread of the virus and has paid less attention to providing care and treatment to individuals already affected. In keeping with past experience in other epidemics, health staff perceived HIV-infected persons as sources of contamination, who should be isolated.

Health care workers are the key providers of medical care. Stigma from health care workers can reduce patients' ability to manage their infection and gain access to health care [7,25]. Persons infected with HIV are often grouped with drug users and sex workers as marginalized, discriminated-against and criminalized elements in society, also by health workers. Stigma towards HIV-infected persons has been documented in health care settings all over the world [26,27]. Showing a negative attitude during counselling and provision of care, treating in a separate area and avoidance of providing service at all are perceived as enacted stigma by HIV-infected patients.

On the other hand, from the health workers' point of view, these actions result from a combination of factors: high workload and personal priority-setting influenced by fear of being infected as well as distrust towards almost all HIV-infected patients because of the association with antisocial behaviors [28,29]. When health care workers have fear and lack knowledge, they can find reasons not to focus their attention on the HIV-positive patients and give those reasons for not providing service as they think/know they should. Moreover, health care workers are not only a source of stigma from the perspective of HIV-infected people, but can also become recipients of stigma from colleagues and family because of their exposure to HIV-infected patients.

The best and most feasible solution is to provide training and reference materials for health workers, to inform them about HIV transmission routes, universal precautions and post-exposure prophylaxis. Reduction of workload would also be important [24]. The results of this study also suggest that the quality of PMTCT service could be enhanced by improving communication and other skills of health workers and providing them with greater support and motivation.

A positive atmosphere in the health facilities should be promoted by normalizing HIV-related services, and undertaking behaviour-change communication campaigns aimed at staff of the health facilities. Feedback from service users could be used as one way to evaluate the quality of service.

In addition, health facilities should make ARV continuously available. The health workers' fear could also be reduced by ensuring that they have and use the protective clothing they need to maintain good hospital hygiene. It will be more difficult to address the issues of fear and stigma towards drug users and sex workers.

Self-help groups of both drug-using and non-drug-using women in Hanoi and other countries were able to improve the relationship and communication between health care staff and patients/clients; peer counsellors and a buddy system led to improvements in the health care provided to and accessed by the women [3,30,31]. Successful examples of this intervention have been documented among clubs for tuberculosis patients [32], alcoholics, cancer patients and patients with chronic illnesses and mental problems [30].

Continuous care and support for HIV-infected mothers after delivery was often not seen as a need to be addressed [12,33]. In practice, the fragmentation of the health care system into specialized vertical pillars including a vertical programme for HIV/AIDS is a major obstacle to providing a continuum of care. Medical treatment, including ARV provision and medications for OI, is increasingly available but is often not accessible to PLHIV because of a weak referral system and social stigma [3,7]. The vertical organization of the health care system and the contradictory mandates between sectors obstruct the effective collaboration and referral between different services that the women and their families need. A lack of multisectoral collaboration is a barrier to effective information exchange for patients between national staff in different facilities [34]. Providing information about topics such as abortion, clean needle exchange programmes and condoms is also politically sensitive in voluntary counselling and testing sites.

The study suggests that information on available services should be made known to health workers. Frequent meetings between different service sites should be organized, with the involvement of high-level health staff that can make decisions, to update information on services available and provide feedback on the quality of the referral system. Development of a practical strategy is needed to strengthen and adapt the referral system to meet the needs of patients. For example, comprehensive services for HIV-infected people should be provided at one service site at district level [2].

As information was collected by means of qualitative methods, the identified factors that lead to their failure to give good quality PMTCT could not be quantified and be representative for the health care worker population in Hanoi.

## Conclusion

In conclusion, the results of this study show that health care workers also face a number of barriers in their efforts to provide good PMTCT services at different levels of the health services in Hanoi. These include a high workload, a lack of equipment and materials, lack of training and skills updating, the common fear of the type of patients who may present with HIV, and little support to improve their performance. These weak points can be addressed by a number of feasible interventions. Results of the study contribute to the picture of the PMTCT programme not only in low-prevalence settings, as in Asian countries, but also in high-prevalence settings with weak health care systems, such as African countries, and may require different interventions to improve the quality of the service.

## Competing interests

The authors declare that they have no competing interests.

## Authors' contributions

TAN, AH, PW, PO and YPN devised the concept protocol. TAN, YPN, PW and PO participated in the data collection. TAN, PW and AH analysed the data. TAN, PO, AH, PW and YPN drafted the manuscript. All authors read and approved the final manuscript.
